# Wrist-Worn Sensor Validation for Heart Rate Variability and Electrodermal Activity Detection in a Stressful Driving Environment

**DOI:** 10.3390/s23208423

**Published:** 2023-10-12

**Authors:** Simone Costantini, Mattia Chiappini, Giorgia Malerba, Carla Dei, Anna Falivene, Sara Arlati, Vera Colombo, Emilia Biffi, Fabio Alexander Storm

**Affiliations:** 1 Scientific Institute I.R.C.C.S. “E. Medea”, 23842 Bosisio Parini, Italy; mattia.chiappini@lanostrafamiglia.it (M.C.); giorgia.malerba@lanostrafamiglia.it (G.M.); carla.dei@lanostrafamiglia.it (C.D.); anna.falivene@lanostrafamiglia.it (A.F.); emilia.biffi@lanostrafamiglia.it (E.B.); fabio.storm@lanostrafamiglia.it (F.A.S.); 2 Institute of Intelligent Industrial Technologies and Systems for Advanced Manufacturing, National Research Council of Italy, 23900 Lecco, Italy; sara.arlati@stiima.cnr.it (S.A.); vera.colombo@stiima.cnr.it (V.C.)

**Keywords:** heart rate variability, skin conductance, stress, arousal, driving

## Abstract

Wearable sensors are widely used to gather psychophysiological data in the laboratory and real-world applications. However, the accuracy of these devices should be carefully assessed. The study focused on testing the accuracy of the Empatica 4 (E4) wristband for the detection of heart rate variability (HRV) and electrodermal activity (EDA) metrics in stress-inducing conditions and growing-risk driving scenarios. Fourteen healthy subjects were recruited for the experimental campaign, where HRV and EDA were recorded over six experimental conditions (Baseline, Video Clip, Scream, No-Risk Driving, Low-Risk Driving, and High-Risk Driving) and by means of two measurement systems: the E4 device and a gold standard system. The overall quality of the E4 data was investigated; agreement and reliability were assessed by performing a Bland–Altman analysis and by computing the Spearman’s correlation coefficient. HRV time-domain parameters reported high reliability levels in Baseline (r > 0.72), Video Clip (r > 0.71), and No-Risk Driving (r > 0.67), while HRV frequency domain parameters were sufficient in Baseline (r > 0.58), Video Clip (r > 0.59), No-Risk (r > 0.51), and Low-Risk Driving (r > 0.52). As for the EDA parameters, no correlation was found. Further studies could enhance the HRV and EDA quality through further optimizations of the acquisition protocol and improvement of the processing algorithms.

## 1. Introduction

Physiological responses while driving are mainly associated with driving stress [1], which is caused by both personal characteristics and environmental conditions (e.g., traffic, weather, road type). Although moderate levels of stress can benefit driver attention [2], excessive levels might influence performance [3], thus leading to a higher risk of traffic violations and car crashes.

Self-reported questionnaires are widely used to measure stress due to behavioral patterns [3]; however, electrophysiological measurements, such as electrocardiographic (ECG) signals and electrodermal activity (EDA), allow the real-time detection of stress by detecting changes in heart rate (HR) and skin conductance (SC) reflecting the sympathetic system responses to stress [4,5]. On that basis, Deng et al. showed that a combination of EDA features was the most representative to detect stress [6]. Additionally, Healey and Picard found higher correlations between stress and EDA features than heart rate variability (HRV) metrics, at least in a car driving simulator scenario [7].

Research evidence on electrophysiological mechanisms and driving simulators also outlined the road users’ ability to detect hazardous traffic situations, preventing potential crashes. This ability, defined as hazard perception, manifests itself at the somatic level even before the subject becomes fully aware of the imminent danger [8], influencing driver decision making in risky contexts with crucial impacts on road safety [9]. In addition, experiments showed that experienced drivers might develop a more intense EDA compared to novices when in danger [10,11], thus proving that driving experience can improve the hazard perception mechanism [12].

Although HRV and EDA features are traditionally obtained by applying surface electrodes on the chest (for ECG recording) and on the volar surface of hands or fingers (for SC measurement), to date, wearable devices with embedded sensors have become widely used to collect physiological signals thanks to their ease of use and wearability. Specifically, detecting HRV and EDA features while driving with a minimally invasive wearable device could ease the evaluation and quantification of stress and risk perception levels in drivers within various and demanding driving scenarios and finally their ability to face hazardous events while driving [13,14]. Despite these advantages, wearable devices may be more sensitive to motion artifacts, such as those induced by steering the wheel or gearing up or down, and less reliable than traditional measurement systems [15].

Empatica E4 system (Empatica, Milan, Italy) is a wrist-worn medical-grade device specifically designed for research purposes and mainly used to collect HRV and EDA features through its photoplethysmographic (PPG) sensors and skin electrodes.

A consistent number of studies focused on assessing the reliability of the E4 sensors to measure HRV and EDA features: there was a preliminary study focused on the validation of the E4 PPG sensor to detect atrial fibrillation [16]; more recent studies tested the reliability of the E4 wristband compared to a gold standard configuration to measure EDA driven by emotional stimuli [17] or in research settings that involved dyadic states [18]; Menghini et al. tested the E4 wristband accuracy and reliability in measuring HRV and EDA features over a few stress-inducing scenarios [19]; at last, Stuyck focused on the validation of E4 performances in estimating HRV in a lab-based context. All these studies agreed that the E4 wristband showed modest reliability in measuring the HRV metrics [19,20], and it often failed to gather accurate data for the EDA features estimation [18,19].

Specifically regarding driving environments and stress detection, a recent study evaluated the performances of both Empatica E4 and Faros 360 (Bittium, Oulu, Finland) wearable medical devices, but no comparison nor agreement between the two tested devices and a gold standard system was provided. Moreover, the direct comparison was performed only on HRV time-domain parameters [21].

Thus, to date, no studies investigated the reliability of Empatica E4 to collect stress measures based on both HRV and EDA metrics in demand-increasing driving scenarios. Given this, the final aim of the present study was to evaluate the accuracy of the E4 wristband device to provide reliable stress metrics in stress-inducing conditions and growing-risk driving scenarios. Moreover, our study focused also on increasing the E4 signal quality, namely introducing robust semi-automatic algorithms for the PPG-derived HRV signal reconstruction and EDA analysis.

## 2. Materials and Methods

### 2.1. Participants

Seventeen healthy volunteers between 25 and 41 years of age were recruited, and each participant provided consent to take part in the experimental procedure. Signals from three participants were discarded for technical problems, two of them due to the Empatica 4 device, one to the gold standard system. Thus, the final sample included 14 participants (five male, nine female; mean age = 33 years, SD = 5.5).

### 2.2. Recording Devices

**Figure 1:**
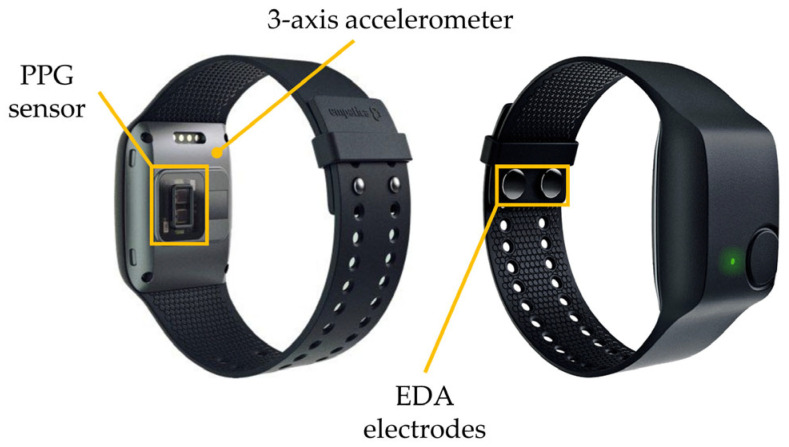
Insight of the relevant sensors positions in Empatica 4.

The tested device was the Empatica E4 wristband (Figure 1), which is a wireless medical-graded wristband able to acquire physiological signals. It includes four sensors: (i) a PPG sensor (LED operation wavelengths: green, with 2 LEDs, and red, with 2 LEDs; 2 photodiodes units, with a total 14 mm^2^ sensitive area) recording variations in the blood volume pulse (BVP) with a sampling frequency of 64 Hz; (ii) two silver-coated stainless steel electrodes with a diameter of 8 mm that record the EDA from the wrist volar surface with a sampling frequency of 4 Hz, a resolution of 900 pS, and an operating range of 0.01 µS–100 µS; (iii) a MEMS-type three-axis accelerometer with a sampling frequency of 32 Hz and with a default range of ±2 g; (iv) an optical frame thermopile to record the skin temperature at 4 Hz.

Participants were asked to wear the E4 wristband on the wrist of their non-dominant hand. Specifically, this choice was made to be consistent with the literature [19,21], provided that there is no difference in using the left or right hand to collect the signals. In fact, during driving scenarios, the steering wheel has to be held with both hands, while the other conditions are potentially motion-free.

The reference system, referred to as the gold standard in the present study, was composed of the following: (i) the eego™mylab amplifier (ANTneuro, Hengelo, The Netherlands), a medical grade device that allows signal collection with an up to 16 kHz sample frequency; (ii) auxiliary and passive sensors (Sensebox XS-271, eemagine Medical Imaging Solutions, Berlin, Germany), connected to the amplifier by means of an adapter, used for collecting the ECG and EDA signals with a sampling frequency of 500 Hz.

The ECG signal was recorded through Ag/AgCl electrodes sized 24 mm in diameter (H124SG, Cardinal Health 200, Waukegan, IL, USA). The ECG electrodes of the reference system were placed on the participant’s chest according to the Lead II configuration: specifically, the negative electrode was placed on the right shoulder, the positive electrode was placed on the left abdomen, and the ground electrode was placed on the right leg.

The gold standard EDA signal was recorded either on the fingers of the non-dominant hand or on the shoulder using Ag/AgCl electrodes. Provided that a slight difference of fingers and shoulder EDA signals was expected and previous studies recommended fingers as the best location for gold standard EDA recordings, the shoulder was also chosen to be an alternative and less noisy acquisition site for driving scenarios. Moreover, some evidence on the strong correlation between the shoulder and fingers EDA signals was reported in [22].

### 2.3. Procedure

Once at the laboratory, participants were informed about the research and, after consent was given, the experimenter positioned the sensors as previously described. Since the EDA signal could not be acquired simultaneously from the two locations, participants were randomly assigned to one of two different groups so to have an equal number of participants that recorded the EDA signal at first on the fingers and then on the shoulder or vice versa. The E4 wristband device was the first device to be placed, thus allowing its sensors to adapt to the participants’ skin; meanwhile, the ECG and EDA electrodes were positioned.

### 2.4. Experimental Conditions

The experimental protocol included six different conditions, which are defined as follows:

- Baseline: Participants were asked to sit and relax for 3 min in front of a black computer screen, keeping their hands on their legs or the armrests and their eyes closed. The researcher also recommended to the participant not to speak and to stay still as much as possible.
- Emotional stimuli, divided into Video Clip and Scream:
  ○ Video Clip: Ad hoc videos eliciting strong emotions (i.e., the fear) and based on a validated database [23] were selected and administered to the participants. Specifically, three scenes, each belonging to a different movie and with a length of 4 min, were edited into a single video clip with a total length of about 12 min.
  ○ Scream: A sudden emotional event composed of a scream accompanied by the image of a frightening face was added at the end of the video clip. Specifically, this condition started a few seconds before the occurrence of the stimulus and lasted about 2 to 3 min. A short break was scheduled after this section to allow the researcher to change the position of the EDA sensors according to the participant’s randomization group.
- Driving videos: The last stage of the experimental protocol consisted of three driving simulations of increasing arousal and potential movement artifacts. The participant was given a steering wheel, a pedalboard, and a gear shifter and was asked to watch the driving videos and follow the main road while simulating the typical gestures of driving (i.e., steering, accelerating, braking, and shifting gears when an acoustic signal was heard). The driving videos had an overall duration of 10 min and 30 s (i.e., 3 min and 30 s per driving video), which were divided as follows:
  ○ No-Risk Driving: The driving scenario depicted a mountain landscape with no potential hazards or risks;
  ○ Low-Risk Driving: It was composed of an accurate selection of several shorter clips extracted from the hazard perception video clips, a British state-of-the-art test for assessing drivers’ skills in risk detection and driving behavior [24];
  ○ High-Risk Driving: This last scenario reproduced potentially dangerous driving simulations, where the drivers’ lack of attention could be harmful.

### 2.5. Data Processing

Raw data collected from the eego™mylab amplifier were exported through the software eego™ (version 1.9.2), while data recorded through the E4 device were saved via the Empatica Connect website into *.csv* files. All data processing was performed in MATLAB (R2022b, The MathWorks, Inc., Natick, MA, USA).

### 2.6. Acceleration Magnitude Signal

Since the E4 device uses a 3-axis accelerometer and provides acceleration data along three orthogonal axes, the acceleration magnitude was computed. Then, this new signal was scaled between ±2 g and filtered with a Butterworth high-pass filter, whose cut-off frequency was set at 0.1 Hz to remove the zero-phase gravitational component.

### 2.7. HRV Analysis

Provided that the duration of each experimental condition was short in time (i.e., from 2 to 4 min), the HRV signal is hereafter to be intended as a short-term HRV signal. A quantitative analysis of the heart rate variability was carried out on the inter-beat interval (IBI) time series. IBIs were computed from the RR intervals of the ECG signal and from the foot points of the BVP signal, respectively. The Pan–Tompkins algorithm [25] was used to automatically detect the R peaks of the ECG signal, while a further visual inspection allowed correcting the ectopic and wrongly detected peaks manually. Regarding BVP, before considering its foot points, the signal was cleaned of the motion artifacts through a third-order Butterworth bandpass filter with subject-specific cut-off frequencies defined according to a fully automated algorithm described below.

At first, the IBI series provided by the E4 device were exploited to detect the BVP artifact-free segments, which were defined as those sequences where the device recorded in real time the IBIs for at least 10 consecutive intervals. For each artifact-free segment, the power spectral density (PSD) estimates of the acceleration module and BVP signals were obtained through the periodogram method [26]. Both PSDs were scaled with respect to their maximum value. Then, the acceleration PSD was subtracted from the BVP analogue; thus, the fundamental frequencies of each artifact-free segment could be extracted from the aggregate PSD and saved only if they were in the 0.5–3 Hz physiological range. The cut-off frequencies of the subject-specific bandpass filter were defined as the minimum and maximum values of the physiological fundamental frequencies, minus or plus an arbitrary safety margin of 0.1 Hz.

The foot points were extracted from the filtered BVP signal through the findpeaks MATLAB (R2022b, The MathWorks, Inc.) function. The wrongly detected peaks were corrected with an interactive user interface, and the continuous IBI sequence was reconstructed for each section of the experimental protocol.

The artifacts detection algorithm proposed by Berntson et al. [27] was applied to both the ECG- and BVP-derived IBIs. Specifically, this algorithm implemented an automatic approach to detect IBIs artifacts, leveraging on the distribution of consecutive heart periods differences. In fact, as suggested by the authors, the beat-to-beat differences generated by artifacts are larger compared to the normal heart period variability; hence, huge differences between consecutive IBIs were used to detect artifacts and classify them into missing beats (i.e., the IBI is approximately twice as large as usual) or extra-beats (i.e., the IBI is approximately half the standard width).

Once the Berntson algorithm was applied, a final visual inspection was performed on the detected artifacts by means of a custom-made user interface to handle false positives or not detected artifacts. Hence, artifacts were replaced with interpolated values from the cleaned IBI series.

Both the ECG- and BVP-derived IBIs were resampled at 4 Hz by means of a piecewise cubic interpolation (i.e., *pchip* MATLAB function), and the Lomb–Scargle periodogram [28,29] was used to estimate their PSDs.

For each experimental condition, according to what was stated in the literature [30], the most relevant time and frequency domain parameters were obtained. On that basis, the average IBI length (Mean IBI, in ms), the standard deviation of IBI time series (SDNN, in ms), and the root mean square of the successive IBIs differences (RMSSD, in ms) were computed in the time domain. In the frequency domain, the normalized spectral contents at low frequencies (LF Spectrum, 0.04–0.15 Hz) and high frequencies (HF Spectrum, 0.15–0.4 Hz) were computed. The very low frequency (VLF) spectral content was not accounted for since, to date, no evidence of the mechanisms underlying the development of VLF components was found [31].

### 2.8. EDA Analysis

To finalize the quantitative analysis on the EDA signals acquired with both the standard electrodes and the E4, some preprocessing was required. At first, both EDA signals were smoothed according to [32] with a moving average filter across a 1-second window, while a z-score normalization allowed standardizing the dataset and increasing the performances of the signal decomposition algorithm, as suggested by [33].

The convex optimization algorithm *cvxEDA* [33] was applied to decompose the raw EDA signals into their tonic and phasic components. Specifically, the *cvxEDA* parameters were set to be homogeneous among every subject, and their values were chosen according to what was reported by [34]. Then, both the E4 and the gold standard EDA signals were split into smaller sections in agreement with the experimental conditions.

Afterwards, an artifact detection algorithm based on the stationary Haar wavelet transform was applied to each EDA section acquired from both measurement systems. Namely, in line with previous studies [35], the wavelet transform coefficients were used to detect those artifact-related parts of the EDA signals, whose evolution was characterized by abrupt changes due to contact losses between the person’s skin and the electrodes or the E4 device, respectively. On that basis, the first-level wavelet detail coefficients were computed from the raw EDA signals and analyzed to detect local outliers, which were defined as those coefficients that were three times greater than the average value across a 1000 s window. The intervals with consecutive outliers were highlighted as potentially artifact related, but only those intervals where the raw EDA was not monotonous were classified as real artifacts. Finally, as suggested in the literature [36], a visual inspection of the artifact-related intervals was performed via a custom-made user interface to accept or reject the artifact detection algorithm suggestions.

At last, the EDA time and frequency domain parameters for both the measurement systems and for each of the experimental conditions were computed. To this purpose, the tonic and phasic components of the artifact-related intervals from the EDA signals were not considered at all. In the time domain, the following set of parameters was obtained [36]: (i) the average value of the z-score normalized tonic component (Mean EDA Tonic); (ii) the frequency of non-specific peaks (NS.EDRs, in #/s); (iii) the normalized area under the curve (NormAUC, in s). Conversely, in the frequency domain, the normalized spectral content in very low frequency (VLF Spectrum, 0–0.045 Hz) and low frequency (LF Spectrum, 0.045–0.15 Hz) were computed [37].

As for the EDA validation, each experimental condition was classified into Shoulder and Finger, according to the recording sites of the gold standard EDA signal.

### 2.9. Statistical Analyses

All the statistical analyses were carried out in MATLAB (R2022b, The MathWorks, Inc.) and SPSS (Version 21, IBM, Corp., Armonk, NY, USA).

At first, since the E4 device recorded IBI data whenever ideal BVP curves were detected, the performance of the wristband device to gather IBI data from the raw BVP signal was evaluated. In particular, a performance metric (in %) was defined as the ratio between the number of beats natively detected from E4 over the total beats detected from the gold standard system. Then, the quality of the reconstructed BVP-derived IBI series was assessed for each condition by comparing the mean number of detected IBIs with the gold standard analogous and by computing the average and standard deviation of the Detection Rate (i.e., defined as in Equation (1)).

(1) $$Detection\;Rate=1-\frac{\left|\#\;beats_{E4}-\#\;beats_{gs}\right|}{\#beats_{gs}}\%$$

Moreover, the average and standard deviation of the Artifact Rate (i.e., defined as the ratio between the amount of detected artifacts and the total number of detected beats) was computed for both the gold standard and E4 IBI series.

As for the EDA quality, instead, the capability of the measurement systems to record EDA phasic activity was investigated for each condition by computing the amount of times the EDA signal was non-responsive in a participant with respect to the total number of participants.

Secondly, the Spearman correlation coefficient (ρ) was computed for each condition and for each HRV and EDA parameter with the aim of assessing the monotonic association between the gold standard and E4 metrics. Spearman’s ρ was selected over the Pearson correlation coefficient since data were rarely normally distributed. According to the ρ values, the correlation was defined as high (>0.9), moderate (0.7–0.9), or low (<0.7). Although the classic Cohen (1988)’s ranking could have been used for this purpose [38], the alternative ranking was preferred since it has already been used in a related study (i.e., see [19]). In addition, a more stringent correlation ranking can partially cope with the small sample size.

Further, a Bland–Altman [39] analysis was carried out for each EDA and HRV parameter among all the experimental conditions. The Bland–Altman charts were built by plotting the differences between the E4 and gold standard parameters against their averages. Moreover, the mean bias (i.e., the mean difference) and the 95% limits of agreement (LOAs), both expressed in their original units of measure, were appended to the charts. Then, the mean bias accuracy was assessed by testing the bias significance at 95% confidence (i.e., with respect to the null hypothesis of a zero mean difference). Although a normal distribution of the differences was rarely achieved, this should not have a meaningful impact on the reliability of the limits of agreement, according to [39]. Finally, whenever the spread of the differences showed a clear trend with respect to the mean values, the statistical approach proposed by [40] to transform the LOAs was applied.

At last, an absolute error defined as the absolute value of the difference between the E4 and gold standard metric was computed for each participant, condition, and HRV parameter. On that basis, the non-parametric repeated-measures Friedman test was performed on every HRV parameter to assess whether the absolute error distributions were different with statistical significance (i.e., 95% confidence) among the experimental conditions (i.e., H_1_: alternative hypothesis). A pairwise comparison post hoc analysis was eventually performed on those parameters where statistical significance was found. Again, the non-parametric approach was chosen over the parametric analogous, since the absolute errors data were barely distributed according to a normal distribution. Namely, 95% confidence Shapiro–Wilk normality tests [41] were performed over the absolute errors for each parameter and condition before carrying out the non-parametric Friedman tests. Due to the poor correlation results of the EDA parameters, no further statistical tests were carried out on the EDA signals.

## 3. Results

### 3.1. Signals Quality

The average native performance of the E4 device to capture IBI data, hence without processing, was about 52.3% (SD = 14.2%). Table 1, instead, resumes the post-processed E4 IBI signal quality compared to the gold standard for each condition.

For each condition, an almost exact correspondence between the E4 and gold standard median numbers of detected IBIs was observed. Additionally, the Detection Rate average values were extremely close to 100%, and their standard deviation was negligible for all conditions except two. Indeed, the Scream and High-Risk Driving conditions were characterized by a slightly lower average Detection Rate compared to the other conditions. Concerning the Artifact Rate, a systematic increase in the average and standard deviation values was identified for each condition when switching from the gold standard to the E4 measurement system. Therefore, the E4 device was more prone to include artifacts into the IBI series that needed to be automatically or manually detected and fixed.

Table 2 shows the relative amount of times the measurement systems failed to capture the EDA phasic activity, thus leading to non-responsiveness, for each condition.

The gold standard EDA signals recorded at the shoulder showed overall lower quality than the ones at the fingers, as expected. However, provided that the quality drop was too high, especially in Baseline, Video Clip, and Scream, only the EDA signals from the fingers gold standard system were considered for further statistical analyses.

### 3.2. Correlation Analyses

**Figure 2:**
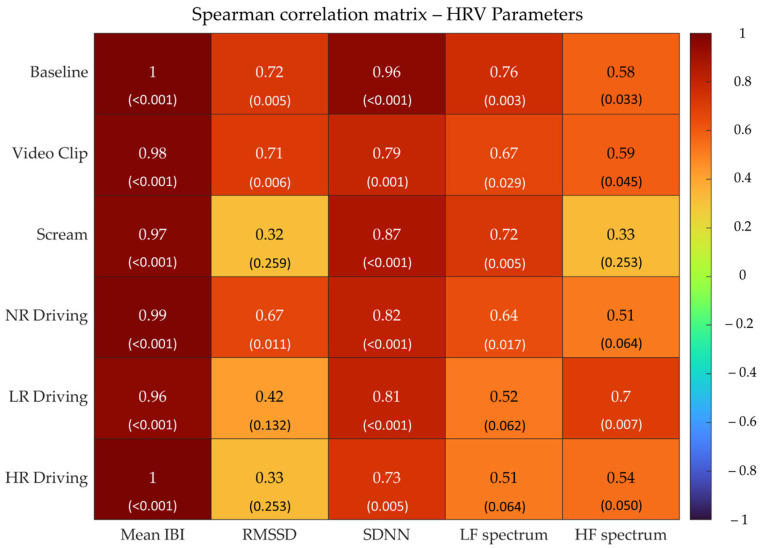
Spearman’s correlation analysis of HRV parameters over six different experimental conditions between the gold standard and E4 measurement systems. *p*-values for the Spearman correlation analysis are reported in brackets.

The Spearman’s ρ values for each condition and HRV parameters are reported in Figure 2.

Overall, Mean IBI showed the highest correlation values for each condition, while for the other parameters, a higher correlation variability among different conditions was observed. Specifically, SDNN exhibited the highest correlation in Baseline, while there were lower but yet moderate correlations in the other five conditions, with High-Risk Driving reporting the worst correlation value for SDNN. RMSSD, instead, showed moderate ρ values in Baseline and Video Clip and low correlation in No-Risk Driving, while no correlation at all was found in the remaining conditions. LF Spectrum reported moderate correlations in Baseline and Screams and low correlations elsewhere. Finally, HF Spectrum showed the best (i.e., moderate) in No-Risk Driving, while low correlations occurred in all other conditions except for Scream, where no correlation at all was found. To sum up, moderate to high correlations for all parameters were estimated in Baseline, moderate correlations were estimated in Video Clip and No-Risk Driving, and lower levels of correlation were estimated in the other conditions.

**Figure 3:**
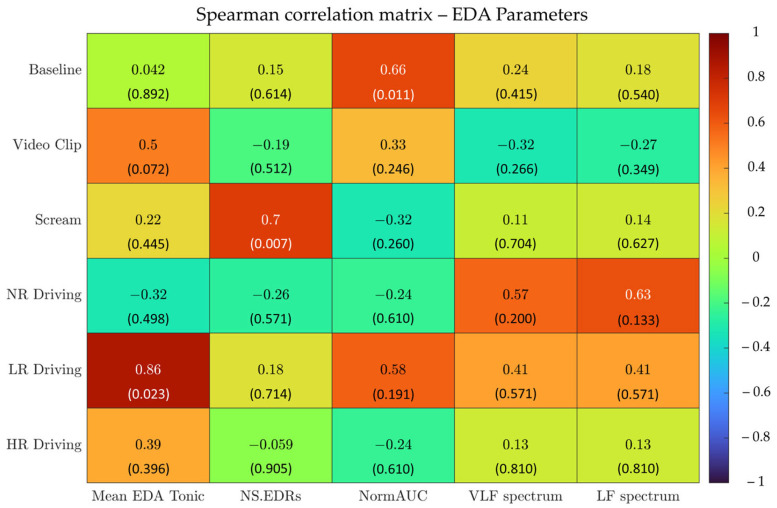
Spearman correlation analyses between the gold standard and E4 measurement systems on Fingers EDA parameters over six different experimental conditions. *p*-values for the Spearman correlation analysis are reported in brackets.

Figure 3 reports Spearman’s ρ correlation coefficients for *Fingers* EDA parameters.

Almost none of the experimental conditions or the metrics show satisfactory correlation results. Moderate correlation values were reported for Mean EDA Tonic in Low-Risk Driving, while low correlations were reported for NS.EDRs in Scream and for NormAUC in Baseline.

### 3.3. Bland–Altman Analyses

**Figure 4:**
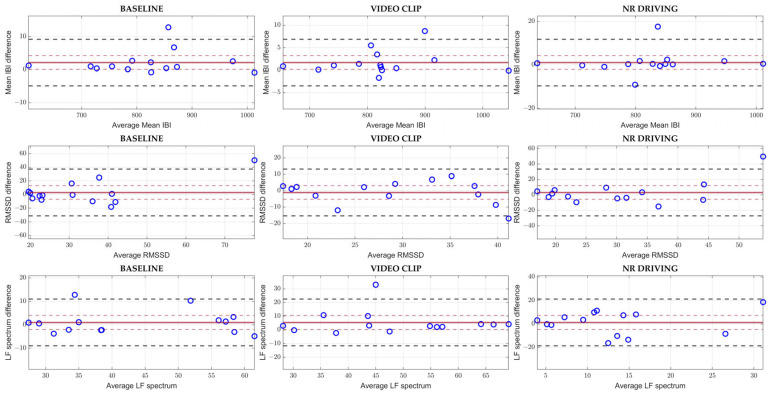
Bland–Altman plots for Mean IBI, RMSSD, and LF Spectrum in Baseline, Video Clip, and No-Risk Driving. The solid red lines indicate the biases, while the dashed red lines stand for the upper and lower confidence interval for the bias values. The dashed black lines represent the upper and lower LOAs. Abbreviations: NR = No Risk.

The Bland–Altman plots for the most representative HRV parameters (i.e., Mean IBI, RMSSD, and LF Spectrum) and experimental conditions (i.e., Baseline, Video Clip, and No Risk Driving) are reported in Figure 4, while the numerical values for biases, confidence intervals, and LOAs are reported in Table 3.

In line with the correlation results, Mean IBI reported the lowest relative differences between the two measuring methods, although a non-null, positive and statistically meaningful systematic bias was observed in Baseline. RMSSD, instead, highlighted non-negligible differences between the two measuring methods as compared to the full scale, especially in Baseline and No-Risk Driving, and the E4 device systematically overestimated RMSSD in the No-Risk Driving condition. Finally, differences between the two measurement systems for the LF Spectrum parameter were limited, especially for Video Clip and Baseline, even though the latter condition reported a positive systematic bias with statistical significance.

**Figure 5:**
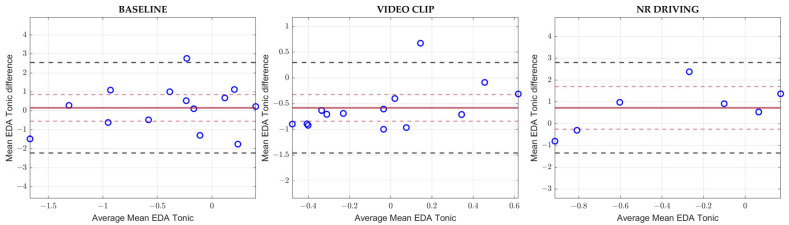
Bland–Altman plots for Mean EDA Tonic in Baseline, Video Clip and No-Risk Driving. The solid red lines indicate the biases, while the dashed red lines stand for the upper and lower confidence interval for the bias values. The dashed black lines represent the upper and lower LOAs.

Figure 5 reports the Bland–Altman plots for the Mean EDA Tonic parameter in Baseline, Video Clip, and No-Risk Driving.

The aforementioned Bland–Altman analysis highlighted the presence of a systematic underestimation of Mean EDA Tonic in Video Clip, and, in general, the limits of agreement were too wide compared to the full scale, showing an almost total disagreement between the two measurement systems, especially in Baseline and No-Risk Driving. Table 3 reports numerical values of the EDA Bland–Altman analysis for biases, confidence intervals, and LOAs. On a side note, the Bland–Altman plots and statistics for each condition and each EDA and HRV parameter are available as Supplementary Materials [42].

Given the poor results of the EDA parameters in every experimental condition, no further statistical analyses were performed to assess the measurement error inter-condition dependence of the E4-derived EDA signal.

### 3.4. Friedman Test

The outcomes of the Friedman test for each parameter are reported in Table 4.

**Figure 6:**
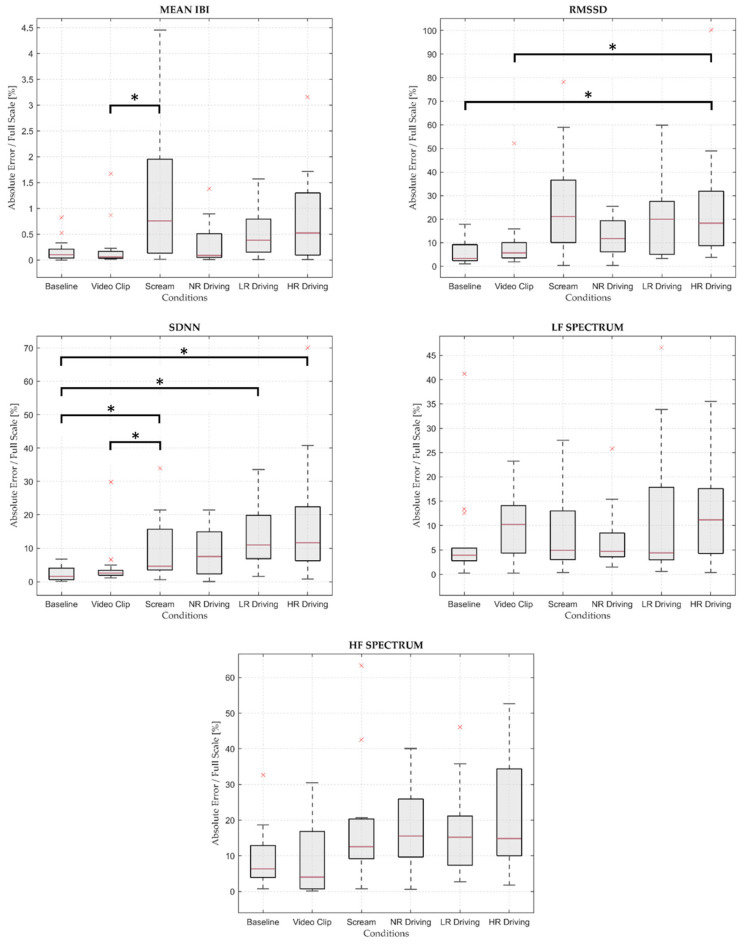
Boxplots reporting the absolute errors distribution for each HRV parameter. Square brackets plus an asterisk indicate statistical significance (*p* < 0.05), according to the pairwise comparison post hoc analysis. The full scale is defined with reference to the Bland–Altman plots for the HRV parameters (Figure 3). Red cross stands for an outlier. Abbreviations: NR = No Risk; LR = Low Risk; HR = High Risk.

The results of the test showed statistically significant differences between conditions for Mean IBI, RMSSD, and SDNN, while no statistically significant differences were highlighted for LF Spectrum and HF Spectrum. On that basis, a pairwise comparison post hoc analysis was performed on the statistically meaningful parameters. Mean IBI highlighted statistically significant differences between Video Clip and Scream (*p*-value = 0.004) and, namely, Scream was characterized by a higher median value than Video Clip. As for RMSSD, the median metrics were higher in High-Risk Driving than in Baseline (*p*-value = 0.004) and in Video Clip (*p*-value = 0.037). Concerning SDNN, the median absolute error was lower in Baseline than in High-Risk Driving (*p*-value < 0.001), it was higher in Scream than in Baseline (*p*-value = 0.026), it was lower in Baseline than in Low-Risk Driving (*p*-value = 0.006), and it was lower in Video Clip than in High-Risk Driving (*p*-value = 0.018). The absolute errors (scaled with respect to the parameters full scale, in %) boxplots for each HRV parameter are reported in Figure 6.

## 4. Discussion

The present study aimed at evaluating the performances of a medical-grade wrist-worn device—the Empatica E4 wristband—to estimate the most relevant stress-related parameters in fear-eliciting and growing risk-driving scenarios. The research study tested the reliability, agreement and correlation of the HRV and EDA metrics collected from the E4 device and gold standard measurement system. The E4 signals were processed with semi-automatic algorithms to increase their quality and usability.

### 4.1. Heart Rate Variability

The semi-automatic algorithm that is responsible for the reconstruction of the E4 BVP signal considerably improved the continuity of the E4 IBI time series, providing a high detection rate for all conditions. Conversely, although the IBI series reconstruction was successful, the HRV parameters correlation was higher in those conditions where no major motion was expected, like in Baseline, Video Clip or No Risk Driving conditions, rather than in Scream, Low- and High-Risk Driving, as proved by the overall correlation coefficients. This was due to how intrinsically the E4 BVP reconstruction algorithm worked since the filtering process leveraged on continuous and long enough IBI sequences. Thus, the longer these sequences, the higher the quality of the reconstructed E4 IBI series. Moreover, provided that the detection of the diastolic BVP foot points was performed on a conditioned and reconstructed version of the BVP signal, we are deeply aware that the BVP reconstruction algorithm we implemented undoubtedly induced interval approximation errors [43]. Despite these concerns, our primal purpose for this study was to try to use 100% of the recorded signal and to not exclude any portion of the raw BVP signals.

Mean IBI reported the highest correlation over each condition. This result agreed with the outcomes of the other E4 device validation studies [19,20], thus confirming that the Mean IBI estimation provided by E4 is reliable and in agreement with the gold standard measurement system, even in case of high-risk simulated driving conditions (i.e., in the presence of motion artifacts). Although a positive and significant bias was observed from the Bland–Altman analysis on the Baseline condition, its absolute value is unquestionably negligible as compared to the Mean IBI full scale. A negligible reduction in the reliability for Mean IBI occurred in Scream, but this was probably caused by the shorter observation length of the IBI series in this specific condition as compared to the others.

Also, SDNN reported moderate to high correlation values for each condition. However, according to the Bland–Altman analysis for SDNN (i.e., see Supplementary Material [42]), a non-negligible and statistically meaningful positive offset was introduced by the E4 device, both in Low-Risk and High-Risk Driving, which was likely due to the intrinsically higher signal-to-noise ratio of a wearable device [15], especially in dynamic scenarios, and also to the randomness increase caused by the peak approximation error [43]. In addition, some dependence on the experimental condition for the absolute error was found, mainly among those conditions where the motion frequency was considerably different, like between Baseline and Scream, Low-Risk and High-Risk Driving (i.e., the absolute error is lower in Baseline, as expected), or between Video Clip and Scream. As a result, the E4-derived SDNN parameter is reliable and in agreement with the gold standard system in conditions with limited motions, while SDNN estimation should be used with caution in case of abrupt and frequent motions (i.e., in high-risk driving scenarios).

As for RMSSD, low to moderate correlation coefficients were found for each condition, hence lower than the SDNN analogous, and this was probably due to its higher sensitivity to the HRV variability. Within different conditions, RMSSD correlation was highly variable, reaching moderate levels in Baseline and Video Clip and low levels in No-Risk Driving. Then, no correlation at all occurred in Low- and High-Risk Driving, and this was mainly due to the high motion frequency within the condition itself, which increased the artifacts-induced intrinsic variability of the E4-derived IBI sequence. A lack of agreement for RMSSD between the two measurement systems, especially in Scream, No-Risk, Low-Risk and High-Risk Driving conditions, was supported by the Bland–Altman results, where a positive, non-negligible and significant bias was reported. Whereas the RMSSD overestimation in No-Risk, Low-Risk and High-Risk driving was induced by the constant motions linked to the intense driving tasks, in Scream, this was probably caused by the abrupt motion of the subject elicited by the frightening face and the audio stimulus, which likely developed artifacts in the IBI sequence. To sum up, the moderate reliability of the E4 device for the RMSSD parameter was reported only in the case of motion-free experimental conditions.

Although compared to other studies with similar experimental conditions [19], the mean artifact rates for E4 were significantly lower, the moderate to low correlation values of the E4-derived frequency domain parameters were mainly affected by the artifact correction procedure. Indeed, removing artifactual IBIs and then re-interpolating the missing values could have induced mismatches between the frequency domain outputs of the two measurement systems [43]. On that basis, experimental conditions with non-negligible movements (i.e., Low- and High-Risk Driving) were affected more than others. As for the Bland–Altman analyses, LF Spectrum showed an overall better agreement than HF Spectrum in each condition: specifically, the best agreement occurred in Baseline rather than in Video Clip or No-Risk Driving, although a systematic and significant positive bias was reported only for Baseline. The HF Spectrum parameter (see Supplementary Material [42]), instead, was underestimated with statistical significance in Baseline, while it was overestimated in Scream and in the driving conditions. This was likely caused by the variability increase for the E4 device due to BVP peak detection approximation error, which led to an unexpected increase in the spectral content in the high frequency band. Finally, it should be noticed that no significant difference in terms of absolute error distribution was reported for the frequency domain parameters. At last, the present validation analysis highlighted that the E4 frequency domain parameters should be used cautiously when dealing with dynamic driving scenarios.

### 4.2. Electrodermal Activity

Unfortunately, as in [17,19], no relevant correlation was reported for the EDA parameters independently of the experimental condition. This could be due to different reasons: firstly, EDA activity collected through wrist-worn devices has been shown in the past to be potentially unreliable [18]. Secondly, quite often, the gold standard EDA signal appeared as non-responsive, lacking in phasic activity, and corrupted by artifacts. Moreover, the Bland–Altman analysis on the Mean EDA tonic parameter critically highlighted no agreement between measurements of the fingers gold standard system and the E4 device, which was likely due to the different skin conductances at the wrist and the fingers caused by the non-negligible difference in sweat glands concentration. However, we cannot exclude that further studies could contradict our results and provide evidence for the use of the E4 device for EDA acquisitions in driving scenarios.

### 4.3. Limitations

The aforementioned results should be interpreted in the light of limitations. At first, the small sample size due to some technical problems in the signal acquisition phase certainly limited the powerfulness of the statistical analyses as compared to other related studies in the literature where the sample size was higher [18,19,21]. Second, the BVP reconstruction algorithm introduced non-negligible interval approximation errors in detecting the diastolic points to build the IBI time series, thus lowering the reliability of some HRV metrics. In addition, the poor reliability of both the gold standard system and the E4 device to collect a fully responsive EDA signal critically affected the EDA parameters validation. Finally, slight inconsistencies on the duration of the experimental conditions could have influenced the HRV parameters’ final outputs from both measurement systems.

## 5. Conclusions

According to the discussed results for HRV, the Empatica E4 device was extremely reliable in estimating Mean IBI in all the experimental conditions, including the driving scenarios. A good reliability was also found for the SDNN estimation, while the RMSSD parameter was hardly computed in the case of non-negligible motions of the participant. As for the HRV frequency parameters, these should be interpreted with extreme care. Conversely, the EDA validation proved that the tested device cannot be assessed as reliable for detecting EDA and estimating its time and frequency domain parameters, thus confirming many other statements from the literature [17,18,19], and the need to collect EDA with other wearable devices. On these bases, further studies need to be conducted to enhance the quality of HRV and EDA signals, considering an improvement of the processing algorithms. Specifically, a more accurate algorithm for BVP peak detection should be implemented, thus limiting the randomness increase in the IBI series and the peak detection approximation errors. At the very last, it is worth exploring further usage scenarios where greater HRV and EDA variations are expected, such as conditions in which the user has a direct control over the scene or is more immersed and engaged, e.g., using immersive virtual reality or a real driving simulator with physical feedback and the possibility of safely testing driving scenarios at various levels of demand (i.e., traffic intensity or different weather conditions).

## Figures and Tables

**Table 1:** Assessment of the E4 IBI signal quality, leveraging on the Detection Rate and the Artifact Rate. Abbreviations: NR: No Risk; LR: Low Risk; HR: High Risk.

Condition	Mean Number of Detected IBIs		Mean Detection Rate (%) (SD)	Mean Artifact Rate (%) (SD)	
	Standard	E4		Standard	E4
Baseline	166	165	99.80% (0.34%)	0.84% (2.38%)	0.64% (0.88%)
Video Clip	378	378	99.58% (0.60%)	0.31% (1.07%)	0.85% (1.58%)
Scream	135	132	98.03% (1.89%)	0.09% (0.22%)	1.07% (2.64%)
NR Driving	203	201	99.18% (2.00%)	0.15% (0.56%)	1.38% (1.85%)
LR Driving	192	192	99.32% (0.83%)	0.42% (1.31%)	3.16% (2.28%)
HR Driving	174	174	98.51% (1.53%)	0.54% (1.08%)	2.9% (2.82%)

**Table 2:** Non-responsiveness of the measurement systems to EDA phasic activity for each experimental condition. Abbreviations: GS = Gold Standard; NR = No Risk; LR = Low Risk; HR = High Risk.

System	Baseline	Video Clip	Scream	NR Driving	LR Driving	HR Driving
Shoulder GS	50%	43%	21%	43%	43%	57%
Fingers GS	29%	0%	7%	43%	43%	29%
E4	36%	14%	7%	43%	14%	14%

**Table 3:** Bland–Altman analyses values for the HRV and EDA parameters reported in Figure 3 and Figure 4. Bold types stand for statistical significance according to the aforementioned criterion and the current *p*-value. Upper and lower LOAs are reported as bias ± SD. Abbreviations: CI = Confidence Interval.

Condition	Bias	Bias CI 95%	*p*-Value (Bias)	Upper LOA	Lower LOA
**Mean IBI**					
**Baseline**	**1.70**	**1.53**	**0.03**	**6.89**	**−3.49**
Video Clip	1.07	3.16	0.48	11.80	−9.66
NR Driving	0.52	3.15	0.73	11.23	−10.19
**RMSSD**					
Baseline	−1.07	4.19	0.59	13.14	−15.29
Video Clip	3.18	8.88	0.45	33.34	−26.97
**NR Driving**	**8.19**	**6.92**	**0.02**	**31.67**	**−15.28**
**HRV LF Spectrum**					
**Baseline**	**5.42**	**5.03**	**0.04**	**22.49**	**−11.65**
Video Clip	1.29	5.85	0.64	21.15	−18.58
NR Driving	−0.79	4.56	0.71	14.68	−16.25
**Mean EDA Tonic**					
Baseline	0.15	0.70	0.64	2.54	−2.23
**Video Clip**	**−0.58**	**0.26**	**<0.001**	**0.30**	**−1.46**
NR Driving	0.72	0.98	0.12	2.80	−1.35

**Table 4:** Non-parametric repeated measures Friedman test results for HRV parameters. Bold types highlight statistical significance.

HRV Parameter	Chi-Squared	Significance	Decision
**Mean IBI**	**18.163**	**0.003**	**Reject H_0_**
**RMSSD**	**21.306**	**0.001**	**Reject H_0_**
**SDNN**	**25.918**	**<0.001**	**Reject H_0_**
LF Spectrum	4.898	0.428	Mantain H_0_
HF Spectrum	10.449	0.063	Mantain H_0_

## Data Availability

Data are available at https://doi.org/10.5281/zenodo.8059242 (accessed on 23 June 2023).

## Supplementary Materials

The following supporting information can be downloaded at https://doi.org/10.5281/zenodo.8059242 (accessed on 23 June 2023).
